# A Random Forest Classifier for Anomaly Detection in Laser-Powder Bed Fusion Using Optical Monitoring

**DOI:** 10.3390/ma16196470

**Published:** 2023-09-29

**Authors:** Imran Ali Khan, Hannes Birkhofer, Dominik Kunz, Drzewietzki Lukas, Vasily Ploshikhin

**Affiliations:** 1 Airbus Endowed Chair for Integrative Simulation and Engineering of Materials and Processes (ISEMP), University of Bremen, Am Fallturm 1, 28359 Bremen, Germany; birkhofer@isemp.de (H.B.); ploshikhin@isemp.de (V.P.); 2 Electro Optical Systems GmbH, Robert-Stirling Ring 1, 82152 Krailling, Germany; dominik.kunz@eos.info; 3 Leibherr-Aerospace Lindenberg GmbH, Pfänderstraße 50-52, 881161 Lindenberg, Germany; lukas.drzewietzki@liebherr.com

**Keywords:** machine learning, random forest, quality inspection, laser powder bed fusion, process monitoring, optical tomography, computerized tomography, gas pores, lack of fusion

## Abstract

Metal additive manufacturing (AM) is a disruptive production technology, widely adopted in innovative industries that revolutionizes design and manufacturing. The interest in quality control of AM systems has grown substantially over the last decade, driven by AM’s appeal for intricate, high-value, and low-volume production components. Geometry-dependent process conditions in AM yield unique challenges, especially regarding quality assurance. This study contributes to the development of machine learning models to enhance in-process monitoring and control technology, which is a critical step in cost reduction in metal AM. As the part is built layer upon layer, the features of each layer have an influence on the quality of the final part. Layer-wise in-process sensing can be used to retrieve condition-related features and help detect defects caused by improper process conditions. In this work, layer-wise monitoring using optical tomography (OT) imaging was employed as a data source, and a machine-learning (ML) technique was utilized to detect anomalies that can lead to defects. The major defects analyzed in this experiment were gas pores and lack of fusion defects. The Random Forest Classifier ML algorithm is employed to segment anomalies from optical images, which are then validated by correlating them with defects from computerized tomography (CT) data. Further, 3D mapping of defects from CT data onto the OT dataset is carried out using the affine transformation technique. The developed anomaly detection model’s performance is evaluated using several metrics such as confusion matrix, dice coefficient, accuracy, precision, recall, and intersection-over-union (IOU). The k-fold cross-validation technique was utilized to ensure robustness and generalization of the model’s performance. The best detection accuracy of the developed anomaly detection model is 99.98%. Around 79.40% of defects from CT data correlated with the anomalies detected from the OT data.

## 1. Introduction

In the late 1980s, additive manufacturing technology emerged as a manufacturing tool for application prototypes [1]. Since then, the AM industry has experienced remarkable growth due to its layer-by-layer manufacturing process, which allows for the production of products with complex shapes and various materials [2]. Hence, it plays an important role in many fields, such as aerospace, manufacturing, and automotive. The AM market’s expected annual growth in the next five years is projected to surpass 20%, as stated in an industrial insight report from Wohlers’s associates in 2020 [3]. Despite significant benefits, quality issues affect the advancement of additive manufacturing technology [4]. One of the key technological challenges to overcome in AM is limited process predictability and repeatability [1].

Metal additive manufacturing techniques using laser powder bed fusion (L-PBF) nowadays provide the highest repeatability and dimensional precision for part production and have thus been extensively investigated in both industry and academia. To manufacture a component, L-PBF methods typically employ the following steps: (1) A layer of metal powder of a specific thickness is placed over the machine’s build plate; (2) a laser beam selectively melts the required region within the powder layer; (3) the build plate slides down, and a fresh layer of powder is put onto the build plate. Layer by layer, this procedure is repeated until the part production is complete. The present approach in AM quality assurance is to analyze the component after it is created using computed tomography, which is extremely costly and time-consuming [5]. According to Seifi et al. [6], statistical qualification of AM components based on destructive materials testing may be unacceptably expensive and take over a decade to complete, which is unfeasible, given the tiny batch sizes and time necessary for manufacturing. If defects could be detected in situ, quality assurance costs in metal AM could be reduced significantly.

Porosity is one of the most important defects to avoid, especially for components that require high tensile strength and fatigue resistance. Porosity in L-PBF components can be caused by inadequate melting (i.e., lack of fusion), pre-existing gas holes in metallic powders from the gas-atomizing manufacturing process, and trapping of gas pores during AM processing [7]. Lack of fusion defects in the laser powder bed fusion process refers to irregular and elongated-shaped anomalies that can vary in size from 50 µm to several millimeters. On the other hand, gas pores in L-PBF are spherical in shape and typically range in size from 5 µm to 20 µm [8]. Process anomalies within a layer, which might yield defects such as pores and lack of fusion defects, are closely related to the occurrence of local temperature changes [9]. Optical monitoring data in the form of intensity recordings can reveal these process anomalies which possibly precede defect genesis. Current monitoring systems however produce huge amounts of data that are typically processed only after completion of the printing process.

The introduction of in-situ process monitoring allows for the tracing of defects throughout the process. Process monitoring may be classified into three categories in principle. The first is melt pool monitoring, which monitors the melt pool and its surroundings. The molten pool’s size and temperature characteristics provide information on the process’s stability and the occurrence of local flaws. The second category examines the entire layer in order to discover defects in various sections of each layer. After scanning, the temperature distribution and surface are observed. The geometric development of the build from slice to slice is considered as the third category [10].

Each of the aforementioned methods generates vast quantities of image data, and the time needed to analyze such large datasets is substantial. Consequently, conducting in-situ data analysis for monitoring purposes in additive manufacturing is currently impractical due to extended processing times. However, a specific branch of artificial intelligence (AI) called machine learning offers a potential solution by enabling rapid and dependable analysis of image data [11]. Process monitoring with the application of ML especially convolutional neural networks (CNN) and random forest classifiers has been utilized successfully for defect detection during the AM process. Baumgarti et al. [2] used in-situ layer-wise images captured by a thermographic camera during the L-PBF process to detect defects using convolutional neural networks. Delamination and uncritical splatters were detected with an accuracy of 96.08%. Grad CAM heat maps were plotted to identify defects. Kwon et al. [12] illustrated the use of CNN for laser power prediction utilizing in-situ layer-wise meltpool images acquired by a high-speed camera during the L-PBF process. The developed CNN model can predict laser power values, which can be utilized to identify problematic positions in AM products without requiring destructive inspections.

ML has grown in popularity in recent years because of its exceptional performance in data tasks such as classification, regression, and clustering [13]. Machine learning is described as computer programming that uses sample data and prior knowledge to maximize a performance criterion [14]. Aside from the traditional application of making predictions through data fitting, the scientific community is exploring new and innovative approaches to integrate ML methods into additive manufacturing. Precise identification, analysis, and prediction of defects hold immense promise in expediting the production of metal AM structures that are both solidly constructed and devoid of defects [15]. Mohr et al. [1] used thermography and optical tomography images for in-situ defect detection during the L-PBF process. A layer-wise OT image is captured using an off-axis CMOS camera, which is similar to the monitoring system utilized in this paper (Section 2.2). CT scans were used to assess the outcomes of OT and thermographic imaging. Only significant defects, such as the lack of fusion void clusters, performed well when compared with the CT data. But for pore detection which is one of the major part defects in additive manufacturing, only 0.7% OT pores and Micro-CT pores overlapped, but 71.4% of thermography anomalies and Micro-CT pores overlapped. For high-quality predictions, ML models require huge training data sets. Due to the high experimental costs, there are restrictions in generating sufficient OT data. As a result, it is ideal to employ a machine learning technique capable of developing an anomaly detection model with a small amount of training data [16]. As a result, there is a need to improve the resolution of the OT system or employ new pore detection approaches using ML techniques for better correlation with micro-CT pores, which is one of the main goals of this research.

The main challenges of developing high-quality machine learning algorithms are Limited data for training, high computational costs, and the lack of generalization to new materials and geometries. The utilization of L-PBF encompasses a wide range of materials and intricate geometries. Nevertheless, the development of machine learning algorithms that can generalize effectively across diverse materials and geometries has a significant challenge. This difficulty arises from the distinct behaviors and characteristics exhibited by each material and geometry, necessitating substantial data and model adaptation. The issue at hand is addressed through the utilization of a traditional machine learning method, specifically the random forest classifier. This choice is made due to its ability to overcome the challenge without necessitating a large volume of training data, unlike more widely used machine learning techniques such as convolutional neural networks [17].

The significance of advancements in data processing algorithms in the field of AM monitoring becomes evident when considering their potential broad impact and applicability. Integrating these algorithms into various monitoring and control systems can enhance process repeatability. This integration can also lead to a reduction in post-processing and non-destructive testing, resulting in cost-effective quality assurance. Conventional quality control methods in L-PBF often involve time-consuming post-processing inspections. However, the utilization of machine learning algorithms can automate the defect detection process by analyzing real-time sensor data and identifying patterns associated with defects [18]. This enables faster and more efficient defect detection, facilitating prompt corrective actions and minimizing the need for extensive post-processing inspections. Ultimately, machine learning offers the ability to swiftly analyze and process in-situ data in L-PBF, thereby enabling accelerated defect detection, real-time monitoring, process optimization, and adaptive control. These advantages collectively contribute to improved efficiency, reduced post-processing requirements, and enhanced overall quality in the L-PBF process [2]. This study aims to contribute to process repeatability and quality assurance through the development of a machine learning algorithm for rapid and reliable anomaly detection leading to defects from monitoring data.

Process invariance and optical noise in the generated OT images make it difficult to identify anomalies. When the amount of data is low for image segmentation random forest technique can be used which is a conventional ML approach. Yaokun Wu and Siddharth Misra [19] demonstrated that RF models outperform neural network approaches in terms of noise tolerance. P. Rajendran et al. [20] also used a random forest classifier to segment brain tumors from MR brain images with an accuracy of 98.37%. In this paper, the focus is on the application of machine learning using optical monitoring data to identify anomalies and validate the detected anomalies using defects obtained using the µCT technique.

## 2. Materials and Methods

### 2.1. Material Data

The experiment is conducted on an EOS M 290 laser powder bed fusion machine (L-PBF). A cylindrical metal specimen is built with a diameter of 10 mm and 15.30 mm in length. It consists of 255 layers with a layer thickness of 60 µm. The powder material used is EOS Titanium Ti64, which has a chemical composition corresponding to ASTM F1472 [21] and ASTM F2924 [22]. For additional details regarding the physical, chemical, and thermal properties of the powder, please refer to EOS GmbH [23]. The volume rate and part density of the powder material is 5 mm^3^/s and ≈4.41 g/cm^3^. The operating gas is argon which has a flow rate of approximately 0.6 mbar. The optics of the OT system are designated so that the camera’s field of view corresponds to the size of the platform.

### 2.2. In-Situ Monitoring by Optical Tomography

**Figure 1:**
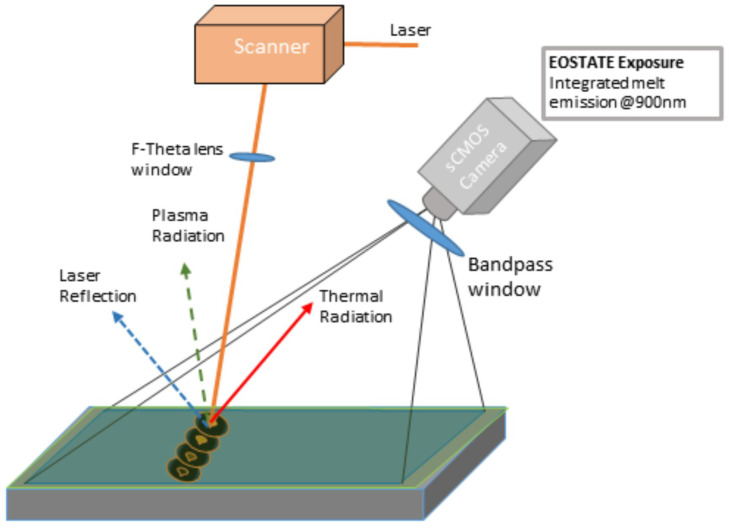
A schematic overview of EOSTATE-Exposure OT system (EOS GmbH).

In-situ monitoring of the L-PBF process is carried out using the optical tomography technique. The OT system used in this study was developed by Electro Optical Systems, (EOS GmbH, Krailling, Munich, Germany) and is known as the EOSTATE-Exposure OT system. During laser powder bed fusion processes powder is melted and three types of emissions are emitted back from the surface such as plasma radiation, thermal radiation, and laser reflection [24]. All radiation with a specified bandwidth is captured by the OT system. Figure 1 shows the schematic overview of the EOSTATE-Exposure OT system. It uses high-resolution CMOS (Complementary metal oxide semiconductor) cameras developed by Exceltas PCO GmbH, which capture signals in the visible and near-infrared spectral range using a band pass filter at 900 nm. The OT system records radiation signals that are proportional to the radiation intensity emitted from the area of the specimen imaged onto the respective pixel element, and are integrated over the entire layer exposition. The basic working principle of this OT system is detailed in [25]. The camera and optics specifications of the OT system are illustrated in Table 1.

**Figure 2:**
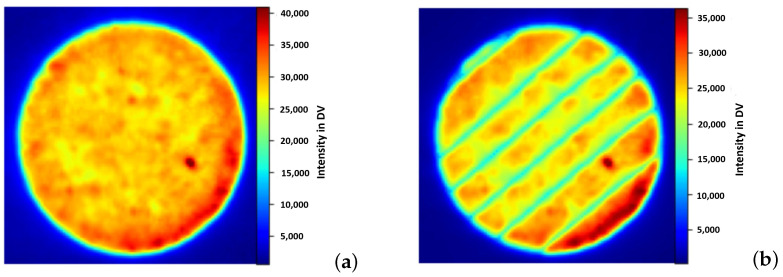
EOSTATE Exposure optical tomography images for the 100th layer under normal process conditions: (**a**) Integral OT image (**b**) Maximum OT image.

**Figure 3:**
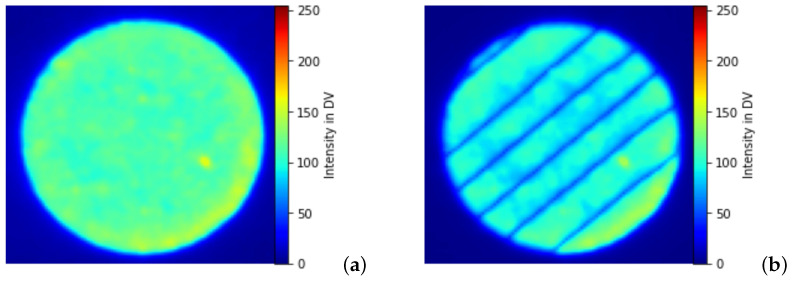
Normalized OT images for 100th layer under normal process conditions: (**a**) Normalized integral OT image (**b**) Normalized maximum OT image.

EOSTATE-Exposure OT generates two types of images: integral gray value images formed by combining a sequence of images (approximately 100) during the platform’s exposure per single layer, and maximum gray value images formed by taking the maximum intensity value of each pixel during the entire layer exposition. Figure 2 shows the integral optical tomography Figure 2a and maximum optical tomography Figure 2b for the 100th layer of the specimen under normal process conditions. The intensity values in Figure 2 are digital values (DV) which are induced by a combination of overlapping scanning strategies, energy increase, and change in temperatures at the building platform. These intensity values range dynamically for different layers and go up to a value of 40,000 DV. Thus to generate a machine learning model it should be normalized to a scalable range [0–255]. Figure 3 shows the normalized integral OT Figure 3a and maximum OT Figure 3b for the 100th layer of the specimen under normal process conditions. These images capture process variances and possible effects of defects. It also helps in analyzing the homogeneity and stability behavior of the build process. Integral OT images are considered for developing an ML model, as featured in these images are more discrete compared to maximum OT images.

### 2.3. Generation of Artificial Defects by Reducing Laser Power

In real-world scenarios, it can be challenging to obtain a sufficient amount of data that contains a wide range of naturally occurring defects. Through the deliberate introduction of defects, a meticulously controlled dataset can be produced, encompassing a diverse range of defect types, sizes, and distributions. This allows for more comprehensive training of the machine learning model. This dataset is instrumental in training a machine learning model capable of accurately identifying both artificially induced defects and naturally occurring defects that may exhibit similar characteristics. Consequently, the model’s capacity to generalize and effectively detect various types of defects in real-world scenarios is enhanced. To summarize, the deliberate introduction of artificial defects in the laser powder bed fusion process proves to be a beneficial strategy for training and assessing machine learning models designed for defect detection [1]. This practice allows for the creation of controlled datasets, enhances the model’s ability to generalize, facilitates accurate performance evaluation, and enables targeted experiments that contribute to a deeper understanding of defect detection in additive manufacturing processes.

**Figure 4:**
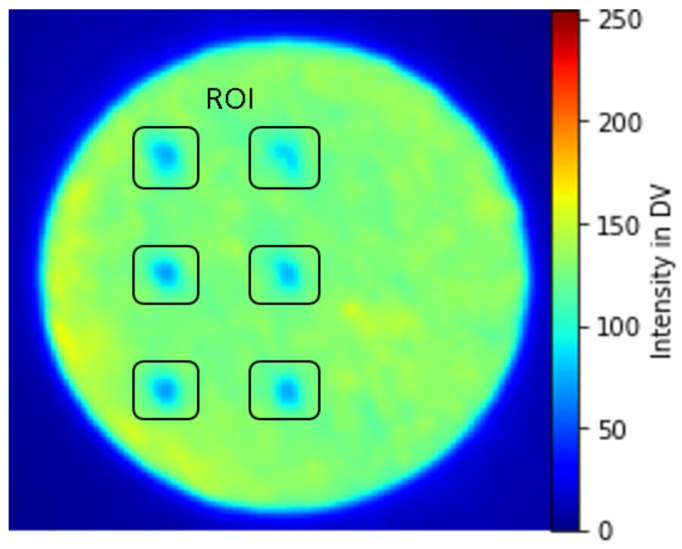
Normalized Integral OT image for layer 101 with induced artifacts.

**Figure 5:**
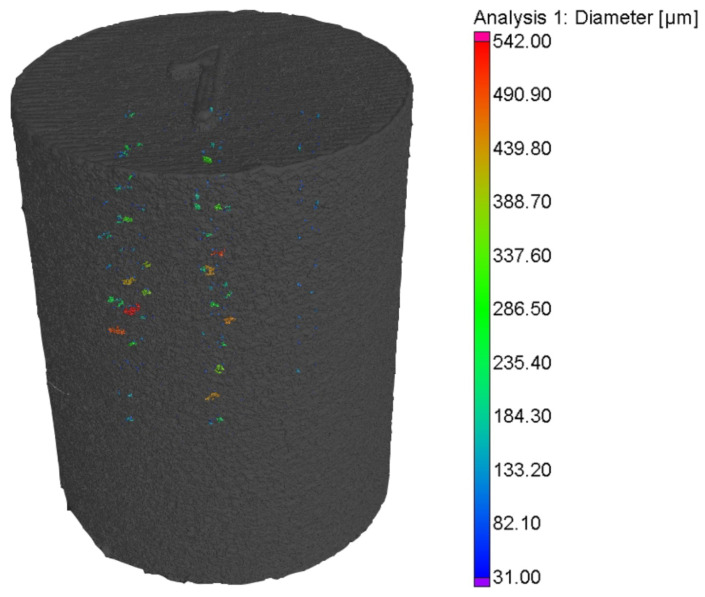
Isometric view of CT specimen along with defects.

In-situ monitoring defects are induced in a cylindrical metal part by applying different processing parameter values at a specific layer height and specific regions called regions of interest (ROI) during the building process. Except for these specific regions, the entire built job is printed with standard process parameters, utilizing a laser wavelength ranging from 1050–1090 µm and a laser beam diameter of 100 µm. At ROI laser power is reduced to 80 watts for four layers, and the laser scan speed and hatch distance are the same as in the standard parameters. Figure 4 shows a normalized integral OT image at the 101st layer height showing ROIs with potential defects due to the reduction of laser power at those specific regions highlighted inside a black box [ROI]. These regions with changes in intensity can be interpreted as anomalies that are caused by changes in temperature and energy density [26]. The entire printed cylinder consists of a total of 8 sections with ROIs in four layers each. Figure 5 shows the isometric view of post-processed CT specimen along with defects caused due to reduced laser power.

The anomalies after detection have to be investigated for potential defects. After completion of the L-PBF process, the built-in cylinders were post-processed and examined using the micro-computerized tomography technique. The majority of defects are gas pores and lack of fusion, ranging from 30 to 540 μm in diameter. An algorithm is developed to correlate anomalies from OT data with defects from CT data to prove the potential of the optical monitoring system in identifying defects during L-PBF processes.

### 2.4. Proposed Model: Random Forest Classifier

Random Forests is an effective machine learning methodology for classification and regression, and it may also be used for image segmentation when training data is limited [27]. RF classifiers have been successfully used for various biomedical image segmentation purposes and this approach can be utilized for defect detection in the additive manufacturing process. Gas pores and lack of fusion, the most critical defects in AM, are considered in this study.

The pre-processing outcomes of optical tomography images revealed that the intensity values of artificially generated defects when normalized, fell within the range of 140 to 170 DV. Interestingly, this range closely aligns with that of non-defect regions, which poses a challenge for image segmentation using histogram-based segmentation, watershed segmentation, or any other direct image segmentation technique. The Random Forest Algorithm is based on the theory of ensemble learning. Ensemble learning is a broad Machine Learning meta-approach that aims to enhance predictive performance by mixing predictions from many models. In layman’s terms, it entails fitting many model types to the same data and then using another model to find the optimum approach to combine the predictions. As a result, the Random Forest Algorithm aggregates predictions from decision trees and chooses the best prediction among those trees [28]. Random Forest is defined as a classifier that comprises some decision trees on various subsets of a given dataset and takes the average to enhance that dataset’s prediction accuracy. Instead of depending on a single decision tree, the algorithm considers the prediction out of each tree and anticipates the ultimate approach that relies on the majority vote of predictions [28].

A Random forest segmentation (RF_Segm) model for pore detection was developed using OT images and ground truth labels. 100 OT images were considered for training the segmentation model. Ground truth labels were generated for these 100 OT images using the Apeer Annotate platform. The segmentation approach consists of two steps: feature extraction and classification of the derived feature vectors for each pixel in the OT image dataset. The Random Forest classifier was trained to associate certain attributes with each pixel in the OT image dataset. The segmentation workflow includes the following sequential steps:

#### 2.4.1. Image Preprocessing

Preprocessing is an important step prior to feature extraction. It includes RGB to grayscale image conversion and image normalization. Due to the dynamic range of intensity distribution of optical tomography images, it is critical to normalize to minimize non-uniform lighting issues. The normalized images are shown in Figure 3. The normalization method determines the mean and variance of an image, reducing the disparity in illumination. Normalization f(x,y) is formulated as in Equation (1) [29]

(1) $$g\left(x,y\right)=\frac{f\left(x,y\right)-M_{f}\left(x,y\right)}{\sigma_{f}\left(x,y\right)}$$

where f(x,y) is original image, $M_{f}\left(x,y\right)$ is the estimation of mean of original image and $\sigma_{f}\left(x,y\right)$ is the estimation of the standard deviation.

#### 2.4.2. Feature Extraction

Feature extraction is the process of establishing a set of necessary features, or image characteristics, that form the core element and, when expressed in an efficient or comprehensible manner, provide the necessary information for analysis and segmentation [30]. A total of 42 feature extractors were generated for training an RF_Segm model. General edge detection operators like Sobel, Prewitt, Roberts, and Canny are used as feature extractors. Other than that Gabor filters, Gaussian blur, median filters, and pixel intensity values of the OT images are used to extract features for generating the segmentation model.

##### Gabor Filter

One of the most well-known feature extraction methods is the Gabor filter. It is made up of wavelet coefficients for various scales and orientations, which makes these features resistant to rotation, translation, distortion, and scaling [31]. In this study, 32 Gabor filters with different orientations and scales were created with a kernel size of 9 × 9. Gabor is a convolutional filter representing a combination of Gaussian and sinusoidal terms. The Gaussian component provides the weights and the sine component provides the directionality. It has excellent localization properties in both the spatial and frequency domains. In the spatial domain, it is a Gaussian-modulated sinusoid, and in the frequency domain, it is a shifted Gaussian. It is represented in Equation (2) [31]:

(2) $$g\left(x,y,\sigma ,\theta ,\lambda ,\gamma ,\phi \right)=exp[\frac{-x^{\prime 2}+y^{\prime 2}\gamma^{2}}{2\sigma^{2}}]exp[i[\frac{2\pi x^{\prime }}{\lambda }+\phi ]]$$

(3) $$x^{\prime }=x\cos\theta +y\sin\theta$$

(4) $$y^{\prime }=-x\sin\theta +y\cos\theta$$

In Equations (3) and (4) *x* and *y* are image coordinates and other parameters which can be varied to generate different Gabor filters are σ, θ, λ, γ, and ϕ. σ is the standard deviation of the Gaussian envelope. θ is the orientation of the filter. γ describes aspect ratio, γ = 1 for circular shape, γ < 1 for elliptical shape. ϕ is the phase offset.

##### Gaussian Blur

The Gaussian blur feature is obtained by blurring an image using a Gaussian kernel and convolving the image. It functions as a non-uniform low-pass filter, preserving low spatial frequency while reducing image noise and insignificant details. A Gaussian function [32] is formulated as in Equation (5).

(5) $$G\left(x\right)=\frac{1}{\sqrt{2\pi \sigma^{2}}}e^{-}\frac{x^{2}+y^{2}}{2\sigma^{2}}$$

where *x* and *y* are the image coordinates and σ is the standard deviation of the Gaussian distribution. A Gaussian kernel with a standard deviation of 3 and 7 is used to generate feature extractors.

##### Edge Detection Algorithms

Sobel, Prewitt, and Scharr are first-order derivative techniques of edge detection that can be used for feature extraction from OT images. The Sobel operator enhances the edges of an image by performing a 2-D spatial-gradient operation on it. The operator is made up of a pair of 3 × 3 convolution kernels that are applied individually to an image to create approximate gradients for each pixel for identifying edges in vertical and horizontal directions. The Prewitt operator finds edges when pixel intensities abruptly fluctuate. It recognizes edges in both the horizontal and vertical axes. Scharr is a filtering method that uses the first derivatives to locate and emphasize gradient edges [33].

##### Median Filter and Pixel Intensity

A median filter was applied to minimize the amount of noise in the stack of two-dimensional OT images. It is a non-linear digital filter used to smooth images which keeps the edges intact. In addition to all of these image filters, the pixel intensity value of each pixel from the OT image is employed as a feature for segmentation.

#### 2.4.3. Training Random Forest Classifier

Compilation of all feature vectors from the extractors for the selected pixels to create the training and testing data set. By including randomness in training samples and combining the output of various randomized trees into a single classifier, the Random Forest addresses the overfitting and generalization problems. The training samples are down-sampled to improve random tree dependency and reduce training time.

**Figure 6:**
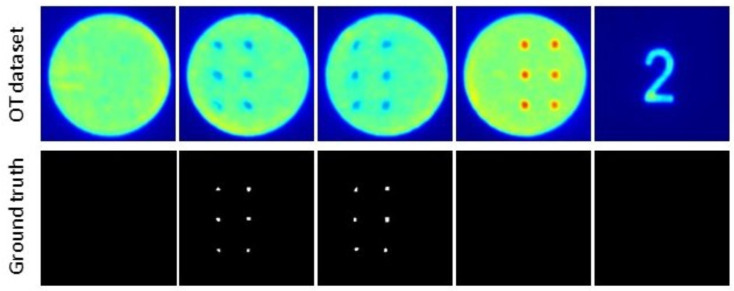
A few OT images and corresponding ground truth labels used in training.

The random forest classifier is trained to identify anomalies from the optical tomography images. Random forest is a pixel-wise segmentation technique where feature extractors are applied on each and every pixel from the OT dataset. A total of 100 images were used for training the model. 100 ground truth labels were manually created by segmenting the anomalies using Apeer Annotate (A free open-source platform from ZEISS) platform. Figure 6 shows the OT images and corresponding ground truth labels for a few images used in the training. The blue-colored regions in the OT images are labeled as anomalies which will be evaluated later with the pores from the CT data. It is evident that the objective is to detect particular blue-colored regions within the image, posing challenges when employing direct image segmentation techniques. The OT images with no anomalies should predict empty gray-scale images. The resolution of a single OT image and mask label is 217 × 217 pixels. So a single OT image consists of 47,089 pixels. As previously described, there are 40 feature extractors, resulting in a total of (47,089 × 40) feature values generated for a single image.

#### 2.4.4. Performance Evaluation on Test Dataset

On the test dataset, the performance of the trained classifier is evaluated. 90% images from the dataset are used for training and the remaining 10% are used for testing. This split is achieved by train_test_split function from sklearn (open source Python library) [34]. The training dataset consists of a total of 47,089 × 90 which is 4,238,010 pixels for training (4,238,010 pixels of OT images and 4,238,010 pixels of mask labels). Similarly, the testing dataset consists of 47,089 × 10 which is 470,890 pixels for testing and validating the developed RF_Segm model. The models are developed for 10, 50, 100, and 1000 estimators to evaluate the prediction accuracy. Training and testing were carried out on the CPU with Intel(R) Xeon(R) CPU E5-1620 v4 @ 3.50 GHz processor with 32 GB RAM.

To ensure that the developed RF_Segm models are not overly dependent on a specific split of the data. Additionally, cross-validation experiments were conducted on one of the RF_Segm models (100 estimators) to ensure the robustness and generalization of the proposed random forest model. The popular K-fold cross-validation technique was used to evaluate the performance of the model in a more rigorous manner. In this approach, the dataset is divided into K equal-sized folds. The model is trained and tested K times, with each fold acting as a test set once and the remaining K-1 folds serving as training. This procedure ensures that the model’s performance is evaluated across many data subsets [35].

### 2.5. Evaluation Using Computed Tomography

Micro-computed tomography is a technique for creating three-dimensional (3D) representations of objects by acquiring multiple X-ray images along an axis of rotation and applying algorithms to reconstruct a 3D model [36]. An industrial 3D micro CT scanner was used to inspect the specimen. Micro CT allows for a comprehensive, non-destructive assessment of the porosity embedded inside AM specimens. The principle of computed tomography is explained in [37]. CT scanning allows for the detection of internal defects in AM parts, including voids, porosity, and cracks. This is achieved by the use of X-rays, which are able to penetrate the part and create a 3D image of the internal structure [38]. The images produced by CT scanning can be used to identify any defects or anomalies in the part. The CT scanner used in this research is located in IABG, Ottobrunn, Germany. It was equipped with a 225 KV micro focus X-ray source and a focal spot size of less than 5 μm and a voxel size of 5 μm with pixel flat panel detector DXR-500L. It has a scanning voltage of 160 kv. The total number of projections is 1440 with a total of 3 frames per projection. The free version of MyVGL which is developed by volume graphics was used to extract layer-wise CT images from the specimens. Additionally, the Register_CT algorithm was created in Matlab programming, employing affine transformation methods to map the coordinates of the extracted CT images onto the optical monitoring images.

### 2.6. Performance Metrics

**Figure 7:**
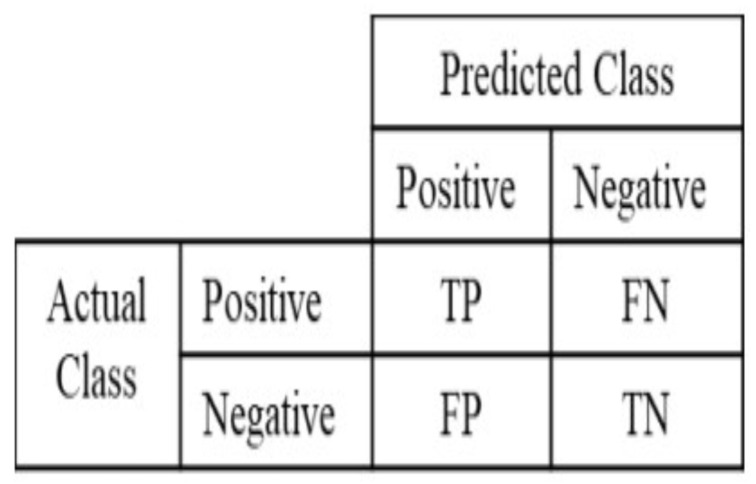
Understanding the confusion matrix [41].

The performances of the RF_Segm model with a varying number of estimators were evaluated using various performance measures, such as confusion matrix, accuracy, dice coefficient, precision, recall, and intersection-over-union. The confusion matrix, often referred to as the error matrix, is represented as a matrix that characterizes how well a machine learning model performs when evaluated on a test dataset as shown in Figure 7.

Where *TP* denotes true positives and is the number of pixels correctly segmented as pores, *TN* denotes true negatives and is the number of pixels correctly segmented as background, and *FP* denotes false positives and is the number of pixels incorrectly segmented as pores. *FN* stands for false negatives and represents the number of pixels that were missed. Accuracy is defined as the proportion of correct estimations to total appraisals. It is concerned with the data set’s quality and defects [39], which is defined as follows:

(6) $$Accuracy=\frac{TN+TP}{TP+FP+TN+FN}$$

The dice coefficient calculates the overlapping pixels between the predicted segmentation pixels and the ground truth pixels as follows [40]:

(7) $$Dice\;Coeff=\frac{2\times TP}{2\times TP+FP+FN}$$

Precision, also known as sensitivity, is defined as the fraction of pore pixels identified as true-positive pixels in relation to all pixels in an OT image classified by the RF_Segm model, which is defined as follows [40]:

(8) $$Precision=\frac{TP}{TP+FP}$$

The recall is calculated as the proportion of true positive pixels classified by the RF_Segm model vs. pixels labeled by manual labeling, and it is expressed as follows [40]:

(9) $$Recall=\frac{TP}{TP+FN}$$

Intersection over Union (IoU), also known as the Jaccard Index, is defined as the area of intersection between the predicted segmentation map *A* and the ground truth map *B*, divided by the area of union between the two maps, and ranges between 0 and 1 [40].

(10) $$IoU=J\left(A,B\right)=\frac{|A\cap B|}{|A\cup B|}$$

## 3. Results and Discussion

### 3.1. Anomaly Detection Using Random Forest Classifier

In this section, the outcomes of employing a random forest classifier for detecting artificially generated anomalies in the L-PBF process are outlined. First, a summary of the performance metrics and prediction time analysis obtained from the random forest segmentation models is presented. This is followed by an elaborate analysis and interpretation of the results. The findings underscore the proficiency of the random forest classifier in anomaly detection during the L-PBF process, elucidating the pivotal factors that impact its performance.

Performance metrics are utilized to assess the effectiveness of RF_Segm models. Four models are developed with 10, 50, 100, and 1000 number of estimators. The performance of developed anomaly detection models on the test data is evaluated using metrics such as Dice coefficient, precision, recall, accuracy, and intersection over union. These metrics are presented in Table 2, which is detailed in Section 2.6. The RF_Segm model with 1000 estimators demonstrated superior metric values. The highest achieved accuracy Equation (6) was 99.98%, indicating the overall accuracy of the model’s predictions. A remarkable IoU score of 71.08 indicated the degree of overlap between the predicted segmentation and the ground truth segmentation. A Dice coefficient of 0.8309 was attained, reflecting the similarity between the predicted and ground truth OT image segmentations. For the RF_Segm model with 1000 estimators, a precision of 0.7705 and a recall of 0.9018 were achieved. These values indicate a minimal number of false negatives compared to false positives, ensuring comprehensive anomaly detection.

**Figure 8:**
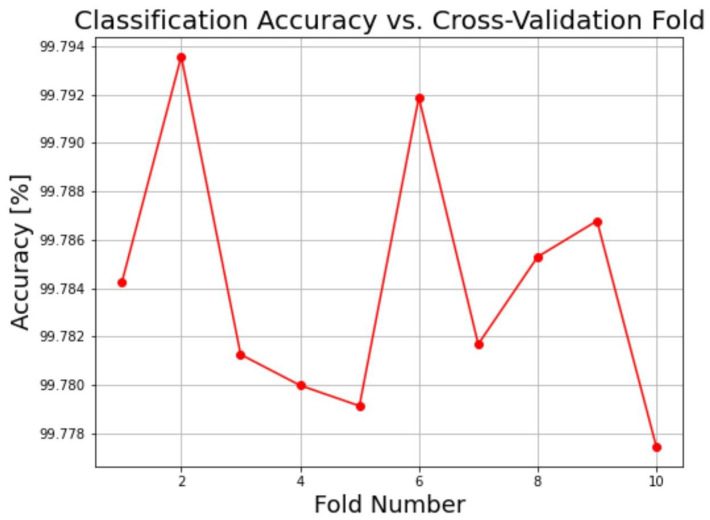
Cross-validation classification accuracy is indicated by the red line across folds for the RF_Segm model with 100 estimators.

The dataset was divided for training (90% dataset) and testing (10% dataset) (Section 2.4.4). To ensure the generalization of the model performance on data splitting, one of the developed anomaly models that is RF_Segm model with 100 estimators was considered for the cross-validation experiment. The data was divided into 10 folds (K = 10), splitting the data into the same shape of 90% training, and 10% testing as used in generating all the anomaly detection models. This approach guarantees that each data point appears in the test set exactly once, reducing the influence of the initial split on model evaluation. Figure 8 shows the plot of metric classification accuracy against each fold. This graph offers a visual depiction of the model’s performance variability across various folds. It illustrates that the model consistently achieves accuracies within the range of 99.77% to 99.79% across all ten folds, affirming its suitability for different data splits.

The confusion matrix (Section 2.6) was calculated for each fold, offering a comprehensive view of the model’s performance in terms of true positives, true negatives, false positives, and false negatives. Specifically, the confusion matrix was computed for the test dataset for each fold, encompassing a total of 470,890 pixels. This approach provides a more accurate evaluation of the model’s performance on the test dataset.

Subsequently, an average confusion matrix was generated by computing the pixel-wise average (mean) of the individual confusion matrices obtained from all 10 folds of the cross-validation. This average representation consolidates the results and offers a comprehensive view of the model’s overall performance.

**Figure 9:**
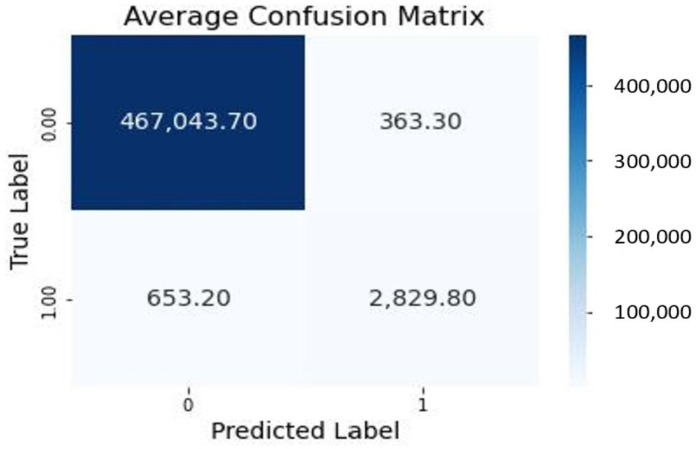
Average confusion matrix: Consistency and performance overview of the RF_Segm model with 100 estimators.

Figure 9 visually presents the matrix representation of the average confusion matrix. In this matrix, ’0’ denotes the number of pixels that do not have any anomalies, whereas ’1’ reflects the number of pixels with anomalies. This illustration provides valuable insights into the model’s performance, allowing us to comprehend its consistency in making accurate and inaccurate predictions, and aids in identifying patterns of errors.

The analysis of the average confusion matrix reveals a predominance of non-anomalous pixels, with a count of ≈467,043 as true positives, accurately identified by the model. Additionally, ≈2829 pixels are correctly recognized as true negatives. On the other hand, there are ≈363 pixels falsely predicted as anomalies (false positives) and ≈653 pixels that are actual anomalies but incorrectly predicted as non-anomalies (false negatives). These numbers highlight the model’s strengths and areas for improvement, providing essential metrics to evaluate its performance.

Further analysis of developed models should consider the detection time to strike a balance between precision and detection speed. The time required for training (90% dataset), testing (10% dataset), and prediction time for a single image of the random forest models with different numbers of estimators are tabulated in Table 3. It can be seen that as the number of estimators gets bigger, so does the time required for training, testing, and anomaly prediction time. The prediction time for an anomaly detection model is of significant importance in the L-PBF process. Low prediction time signifies the timely identification of anomalies, improves process efficiency, minimizes costs, enables real-time monitoring, optimizes resource allocation, and facilitates scalability. These combined factors result in improved productivity, decreased defects, and enhanced quality control within L-PBF manufacturing. A prediction time of 40 ms was achieved for the model with 1000 estimators. This detection time goes better with the performance metrics of the RF_Segm model with 1000 estimators when compared to other developed models. Further, if the number of estimators is increased, a point of diminishing returns is reached. At this point, a marginal improvement in performance becomes smaller and does not justify the additional computational resources and time required for training and predicting anomalies.

**Figure 10:**
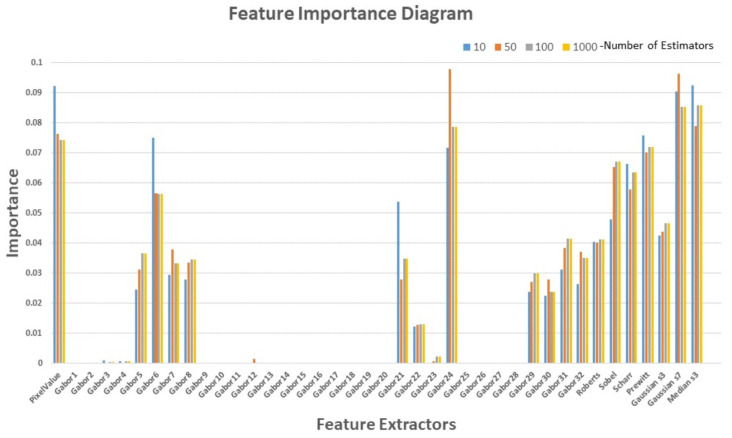
Feature importance diagram for a different number of estimators.

In all the generated ML models, a total of 40 feature extractors were utilized in constructing the RF_Segm models. The importance of each feature and the selection of optimal features for model training were deemed crucial. This process, known as Feature Selection in machine learning, involves the removal of less relevant features, thereby simplifying the model, reducing overfitting, and improving computational efficiency [42]. Feature selection based on feature importance contributes to enhancing the model’s performance and interpretability. The feature importance diagram, as depicted in Figure 10, illustrates the relative importance of each feature for different estimators. This diagram offers valuable insights into the significance of individual features in the segmentation of anomalies in OT images. Notably, the original pixel values of OT images, Gaussian filter, Median filter, and Gabor24 feature extractors exhibit the highest importance values, indicating a strong relationship with the segmentation label. Overall, the feature importance diagram in the random forest segmentation model provides valuable insights for feature selection, understanding data relationships, model interpretation, error detection, and guidance for future data collection endeavors.

**Figure 11:**
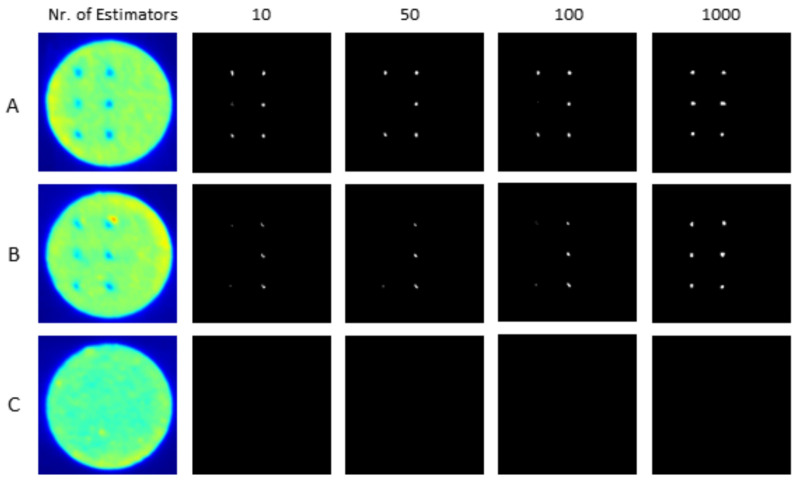
Anomaly prediction in sample optical tomography images (**A**–**C**) utilizing RF_Segm models with diverse estimator counts.

Figure 11 shows the anomaly prediction from OT images for different RF_Segm models developed with different numbers of estimators. In Figure 11, Images A and B are the OT images with artificially induced anomalies and image C is the OT image under normal process conditions. It can be seen that the RF_Segm model with 1000 estimators gives better prediction with respect to models with a lesser number of estimators.

### 3.2. Correlation of OT Anomalies with CT Defects

The anomalies detected by the RF_Segm model were evaluated with defects detected in the CT data. Examination of the CT scans revealed the presence of gas pores and a lack of fusion defects in the printed cylinder. These defects exhibited sizes ranging from 5 to 300 µm. Based on their sizes and shapes, the defects were categorized accordingly. Defects with a spherical shape and sizes below 20 µm were designated as gas pores, while irregularly shaped defects measuring between 20 µm and 300 µm were classified as lack of fusion defects [15].

The correlation of OT anomalies and CT defects is carried out using image registration techniques. This technique reduces spatial ambiguity and enables data comparison. Data from different imaging modalities that had varying acquisition setups and spatial resolutions, particularly in the build direction, were overlaid using image registration. It was crucial to emphasize that, in comparison to CT data, which were gathered after the production process, optical monitoring data were acquired during the printing process. Because of this OT data does not account for shrinkage or other deformations that occur after the completion of the building process [1].

An essential part of the registration procedure was the transformation selection. It determines how a certain image is deformed to match the shape of another image dataset. The affine transformation was used to map CT data image coordinates to the OT data image coordinates system. Affine transformation is a type of geometric transformation that combines translation, rotation, similarity, and shear mapping. The developed image registration algorithm called Register_CT as explained in Section 2.5 was used to register the CT image dataset onto the OT monitoring dataset.

**Figure 12:**
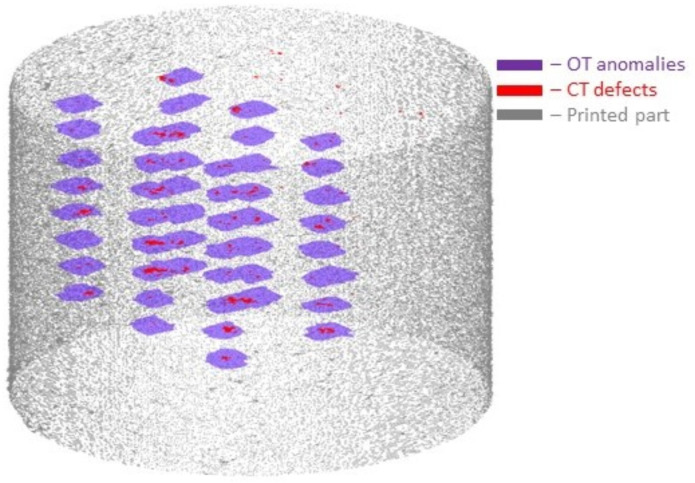
3-Dimensional rendered surface with overlap of CT defects with detected anomalies.

Following the successful registration of CT defects onto the OT images, an algorithm was devised for 3D reconstruction using the mapped dataset. The surface rendering module in Matlab was employed to generate a visual representation of the printed cylinder. Figure 12 displays the 3D rendered topography, illustrating the mapping of CT defects onto the OT anomalies. In the figure, blue regions indicate OT anomalies, red regions depict CT defects, and gray color represents the outer geometry of the printed cylinder. Remarkably, approximately 79.4% of CT defects overlapped with the OT anomalies, indicating a strong correlation between the two datasets.

## 4. Conclusions

In conclusion, the implemented conventional ML algorithm indicates outstanding abilities in detecting process anomalies within the specified range of intensity values. The experiment was carried out using an EOS M 290 L-PBF machine using EOS Titanium Ti64 as material. Random forest segmentation models were created for a variety of estimators, including 10, 50, 100, and 1000. The RF_Segm model with 1000 estimators obtained an astounding 99.98% accuracy while keeping a fast prediction time of 40 ms. The reported instabilities were analyzed using defects identified using the CT approach to test the algorithm’s robustness. 79.4% of the defects identified in the CT data correlated with the anomalies reported by the optical monitoring system, which is promising. This finding emphasizes the proposed random forest segmentation model’s potential for quality inspection during L-PBF procedures, outperforming current CT correlation standards in in-situ anomaly identification.

Furthermore, the developed model showcases remarkable efficiency in terms of computational costs, which stands as a significant advantage in utilizing the random forest classifier for anomaly detection model development. Despite the limited training data, consisting of only 100 OT images with corresponding ground truth labels, a segmentation model with an accuracy of 99.98% was successfully created. The model’s training process also offers a notable advantage in terms of time requirements. Merely approximately 3 h of computational training was necessary to construct the RF_Segm model with 1000 estimators. This aspect enhances its efficiency and ensures optimal utilization of resources. It is worth highlighting that the model effectively identifies artificially induced defects with reduced laser power parameters and establishes a correlation with defects detected in the CT data.

This paper presents the successful detection of anomalies utilizing the RF_Segm model in the context of in-situ anomaly detection in L-PBF. The anomalies detected in this study were subsequently evaluated and identified as gas pores and lack of fusion defects using the CT technique. These two types of defects are known to significantly impact the fatigue life of printed parts in the L-PBF process. By effectively identifying and characterizing these critical defects, the RF_Segm model contributes to quality assurance and reliability improvement in additive manufacturing processes. The findings of this study highlight the potential of the developed model in enhancing the overall structural integrity and performance of L-PBF-produced components.

In summary, the developed random forest segmentation model, integrated with the optical monitoring system, exhibits exceptional accuracy, swift prediction time, and strong correlation with CT data. Its potential for quality inspection during L-PBF processes demonstrates its efficacy in detecting anomalies and ensuring manufacturing integrity. Further research and validation on larger datasets are warranted to fully exploit the model’s capabilities and advance anomaly detection in L-PBF processes.

## 5. Concluding Remarks

The research has demonstrated the effectiveness of machine learning algorithms in the realm of anomaly detection, particularly in the context of EOS Titanium Ti64 produced by the EOS M 290 L-PBF machine. The developed ML algorithm has showcased remarkable performance, achieving an accuracy rate of 99.98% in identifying anomalies within specified intensity ranges. Notably, it outperforms conventional CT standards, underscoring its potential for enhancing quality assurance processes in the additive manufacturing industry.

Part defects such as gas pores and lack of fusion defects were successfully identified through CT data analysis. which was further correlated with detected anomalies which gave a remarkable correlation accuracy of 79.4%. This underscores the promising capability of optical monitoring systems in enhancing the quality assurance procedures for laser powder bed fusion processes.

Looking ahead, our focus is on the future prospects of integrating machine learning with optical monitoring techniques to further enhance quality assurance in L-PBF processes. Envisioning the utilization of CNN models for faster anomaly detection, harnessing a comprehensive dataset of over 2000 real-time images. Our ongoing efforts will be directed toward improving model robustness and enhancing detection accuracy, paving the way for more reliable and efficient quality control in additive manufacturing.

## Figures and Tables

**Table 1:** Technical specifications of the EOSTATE-Exposure OT system.

Specifications	Values
Spectral range	887.5 nm–912.5 nm
Camera resolution	2560 × 2160 pixels
Objective lens	8 mm
Frame rate	10 fps
Spatial resolution	125 µm/Pixel
Data interface	USB 3.1

**Table 2:** Performance metrics for RF_Segm model with different number of estimators.

Number of Estimators	Dice Coeff	Precision	Recall	Accuracy [%]	IOU Score
10	0.7068	0.6489	0.7760	96.96	54.66
50	0.7952	0.7334	0.8660	97.96	65.87
100	0.8200	0.7604	0.8899	99.67	69.50
1000	0.8309	0.7705	0.9018	99.98	71.08

**Table 3:** Time required for Training and Testing the RF_Segm model.

No. of Estimators	Training (sec)	Testing (sec)	Prediction Time/Image (sec)
10	100	01	0.011
50	501	02	0.012
100	1115	04	0.014
1000	10,564	35	0.04

## Data Availability

The data presented in this study are available on request from the corresponding author. The data are not publicly available due to restrictions from the project partners.

## Abbreviations

The following abbreviations are used in this manuscript:

AM	Additive Manufacturing
OT	Optical Tomography
ML	Machine Learning
µCT	Micro-Computerized Tomography
EOS	Electro-Optical Systems
IABG	INDUSTRIE ANLAGEN-BETRIEBS GESELLSCHAFT
IOU	Intersection-Over-Union
L-PBF	Laser Powder Bed Fusion
CNN	Convolutional Neural Network
CMOS	Complementary metal-oxide-semiconductor
DV	Digital Values
ROI	Regions Of Interest
RF_Segm	Random Forest Segmentation
RGB	Red Green Blue
3D	Three Dimensional
KV	Kilo Volts
TP	True Positives
TN	True Negatives
FP	False Positives
FN	False Negatives
µm	Micrometer
ms	Millisecond
